# Commentary: Association Between Antihypertensive Medication Use and Breast Cancer: A Systematic Review and Meta-Analysis

**DOI:** 10.3389/fphar.2021.732622

**Published:** 2021-08-05

**Authors:** Natansh D. Modi, Ahmad Y. Abuhelwa, Bradley D. Menz, Ross A. McKinnon, Michael J. Sorich, Ashley M. Hopkins

**Affiliations:** ^1^ College of Medicine and Public Health, Flinders University, Adelaide, SA, Australia; ^2^ Division of Pharmacy, Southern Adelaide Local Health Network, Flinders Medical Centre, Adelaide, SA, Australia

**Keywords:** commentary articles, concomitant, breast cancer, immunotherapies, beta-blocker

## Introduction

In an emerging age of precision medicine, clarity on the impact of concomitant non-cancer medicines on the efficacy of anti-cancer treatments has never been so important. For example, immunotherapies are emerging as an important treatment option in breast cancer (e.g., atezolizumab, pembrolizumab) (Schmid et al., 2018; Cescon et al., 2020). Further, it is hypothesised that concomitant medicines such as beta-blockers, statins, and metformin may boost immunotherapy actions (Kokolus et al., 2017; Afzal et al., 2018; Cortellini et al., 2020), while antibiotics and proton pump inhibitors may impair responses (Hopkins et al., 2020a; Hopkins et al., 2020b; Cortellini et al., 2020). However, undertaking randomised control trials to identify the impact of each non-cancer concomitant medicine on breast cancer prognosis is not practicable due to cost, exposure to potentially harmful strategies, and years until results become apparent. Thus, we read with great interest the systematic review and meta-analysis from Xie et al. on the association between concomitant antihypertensive medication use and breast cancer risk and prognosis (breast cancer-specific mortality, recurrence, overall survival, and disease-specific survival) (Xie et al., 2021). Xie et al. (2021) concluded that the use of calcium channel blockers, beta-blockers, or diuretics was significantly associated with an increased risk of developing breast cancer, and that diuretic use may elevate the risk of breast cancer-specific mortality (with no statistically significant association found for calcium channel blockers, beta-blockers, or renin-angiotensin system inhibitors). Underlying assumptions of these conclusions are that the meta-analysis strategy acquired sufficient power to assess the associations and the author team adequately evaluated and filtered original scientific literature for quality. In relation to this evaluation, we appreciate the scoring system used by the authors to differentiate the quality of prior studies, however greater detail on the specifics of each study achieving their score would be appreciated.

## Type of Data

Evaluating the association between concomitant antihypertensive use and survival outcomes in breast cancer has been undertaken using multiple data types, including observational, health registry, electronic health record (EHR), and clinical trial data. Each data type has strengths and weaknesses for providing affordable, rapid, hypothesis-generating findings. For example, health registries and EHR are often very large and matched to contemporary practice, but may have limited value with regards to treatment, comorbidity, cancer subtype, and other adjustment data to extract causality insights (as compared to some trial datasets) (Mack et al., 2018). As such, it would be useful if Xie et al. (2021) provides clear information on the data types used in each meta-study and whether results differ according to registry/EHR versus clinical trial data.

## Immortal Time Bias

Immortal time bias may greatly inflate the apparent association between an exposure (e.g., beta-blocker use) and survival in observational studies that investigate an exposure that is not known at the start of the survival follow-up period (e.g., at cancer diagnosis) (Lévesque et al., 2010). If individuals who commence the use of a beta-blocker at a later time are included in the exposed group, then it is important to use more complex analysis methods (e.g., landmark, time-dependent covariate) to avoid immortal time bias (Weberpals et al., 2016).

We note that Cardwell et al. (2014) and Cui et al. (2019) provide explicit statements on their methodologies to account for immortal time bias (nested case-control and time-dependent approach respectively). We would appreciate it if Xie et al. (2021) could provide information on whether each study used methodologies to mitigate immortal time bias, and whether meta-analysis results differed between studies that did and did not account for immortal time bias.

## Influence of Stage, Subtypes, and Therapy

We advocate that analyses performed to identify the association between antihypertensive use and survival outcomes in breast cancer need to differentiate between subtypes (e.g., HER2 positive, triple-negative), stages of disease (early vs. advanced), and lines of therapy. For example, Modi et al. (2020) identified worse overall survival amongst patients using beta-blockers and treated with contemporary trastuzumab, pertuzumab, ado-trastuzumab emtansine, and docetaxel therapies for HER2 positive advanced disease. In contrast, Spera et al. (2017) identified improved progression-free survival amongst patients using beta-blockers and treated with contemporary ramucirumab and docetaxel therapies for HER2 negative advanced disease.

These opposing results demonstrate findings may be biologically different between breast cancer subtypes, stages, lines of therapy, or treatment options and that future evaluation of the associations between antihypertensive use and outcomes in subgroups would be valuable.
